# Metacommunity versus Biogeography: A Case Study of Two Groups of Neotropical Vegetation-Dwelling Arthropods

**DOI:** 10.1371/journal.pone.0115137

**Published:** 2014-12-30

**Authors:** Thiago Gonçalves-Souza, Gustavo Q. Romero, Karl Cottenie

**Affiliations:** 1 Programa de Pós-Graduação em Biologia Animal, Departamento de Zoologia e Botânica, IBILCE, Universidade Estadual Paulista, UNESP, São José do Rio Preto, SP, Brasil; 2 Departamento de Biologia Animal, Instituto de Biologia, Universidade Estadual de Campinas (UNICAMP), Campinas, SP, Brasil; 3 Department of Integrative Biology, University of Guelph, Guelph, ON, Canada; CNRS, University of Montpellier II, France

## Abstract

Biogeography and metacommunity ecology provide two different perspectives on species diversity. Both are spatial in nature but their spatial scales do not necessarily match. With recent boom of metacommunity studies, we see an increasing need for clear discrimination of spatial scales relevant for both perspectives. This discrimination is a necessary prerequisite for improved understanding of ecological phenomena across scales. Here we provide a case study to illustrate some spatial scale-dependent concepts in recent metacommunity studies and identify potential pitfalls. We presented here the diversity patterns of Neotropical lepidopterans and spiders viewed both from metacommunity and biogeographical perspectives. Specifically, we investigated how the relative importance of niche- and dispersal-based processes for community assembly change at two spatial scales: metacommunity scale, i.e. within a locality, and biogeographical scale, i.e. among localities widely scattered along a macroclimatic gradient. As expected, niche-based processes dominated the community assembly at metacommunity scale, while dispersal-based processes played a major role at biogeographical scale for both taxonomical groups. However, we also observed small but significant spatial effects at metacommunity scale and environmental effects at biogeographical scale. We also observed differences in diversity patterns between the two taxonomical groups corresponding to differences in their dispersal modes. Our results thus support the idea of continuity of processes interactively shaping diversity patterns across scales and emphasize the necessity of integration of metacommunity and biogeographical perspectives.

## Introduction

Since the early development of the ecological theory, understanding the mechanisms that drive small- and large-scale patterns in species richness and composition received primary interest [1]–[4]. The relative importance of local (e.g., species interactions such as predation and competition) and regional processes (e.g., dispersal, speciation) in explaining the diversity patterns generated much discussion in the last 30 years. The initial argument was that local processes determine diversity patterns, but the pioneer studies of Robert MacArthur emphasized that regional processes could also drive small- and large-scale diversity patterns [5], [6]. The proponents of these two point of view established hot debates that contributed to important advances to the ecological theory. It has been now suggested that a balance between local and regional processes govern species diversity at both small and large scales [6]–[8]. For instance, Cornell and Harrison [9] argued that there is a continuum of processes operating more or less intensely from small to large scales [10]. As a result, local interactions and dispersal constitute processes working together to assemble communities [10], and thus local and regional processes are both important [11]. This interaction between local and regional processes and their effects on community structure at different scales are explicitly tested in metacommunity theory, which considers a set of local communities linked by dispersal of potential interacting species [12]. However, there is at least one other conceptual scale above the metacommunity: biogeography. Biogeography explains patterns at bigger spatial and temporal scales, often including evolutionary processes [5]–[7]. The confusing part is that often the same types of processes are used to explain metacommunity and biogeography patterns, such as niche differentiation and dispersal limitation.

If geographical distance among different localities limits the dispersal of organisms, and thus imposes for instance range limits on species independent of environmental variation, compositional similarity will thus be spatially structured at biogeographical scale [12]–[14]. On the other hand, at the metacommunity scale, assemblages are often environmentally structured because niche-based processes such as microhabitat type generally cause strong differences in local demography of species that, in turn, affects local species composition (species sorting perspective) [12]. These predictions of dispersal- and niche-based perspectives are not mutually exclusive [13]. Recent works suggest that the relative importance of niche- and dispersal-based processes may change from small to large scales [15], [16]. For instance, Márquez and Kolasa [17] experimentally demonstrated that niche-based processes assemble local communities, but their strength depended on other factors such as dispersal. Thus, empirical studies are still necessary to understand the ways in which these processes contribute to (interactively) affect communities at different scales.

Jocqué *et al*. [18] have explicitly integrated processes acting at different scales to understand patterns of community structure. These authors suggested a trade-off between dispersal (a regional/biogeographical process) and species' ecological specialization to local conditions as an important driver of large-scale diversity patterns [19], [20]. In that study, Jocqué et al. [18] derived three predictions: first, that ecological specialization limits dispersal, since the chance of colonizing suitable habitats for locally specialized species decreases away from the optimal habitat. Second, that longer dispersal distances will be present in more climatically variable environments, since this allows organisms to follow their optimal habitat conditions [19]. Third, that higher level of endemism will be present in more stable environments because of higher speciation rates. The framework suggested by Jocqué et al. [18] adopts an important recommendation from Weiher et al. [21] in which metacommunity ecology and biogeography should be integrated to disentangle the relative importance of multiple processes acting to assemble ecological communities. In addition to providing these clear predictions, Jocqué *et al*. [18] also implicitly explore the dual nature of niche and dispersal processes at either metacommunity and biogeography scales. At biogeographical scale, niche and dispersal processes are linked through evolutionary trade-offs, while at the metacommunity scale sensu Leibold et al. [12], these evolutionarily determined niche and dispersal traits are exposed to actual local communities of species interacting with each other and their environment, dispersing at different rates throughout the landscape based on connections between the different sites etc.

**Figure 1:**
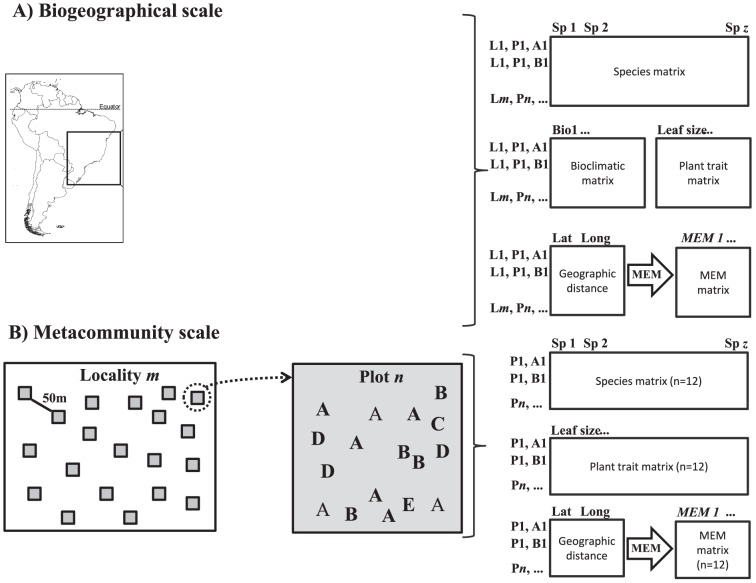
Multiple scales used in the study. A) Map of South America (left) and the geographical range of the study (middle). The symbols present each 12 sampled localities; localities with similar symbols (grey squares, black triangles and grey circles) have similar climatic characteristics (Fig. S1 in S1 Appendix). From Northeast to South, the order of the sampled localities is the same as in Table 1. Each row of the local matrices (n = 12 per arthropod group) presents the sampled plot (P*n*) and individual plant (A, B, C, D or E_1 to 20_) (1B, right). At the biogeographical scale (1A, middle), we used a species matrix (including all localities), two groups of environmental variables (climate and plant architectural features), and the distance among plots to perform the RDA_biogeographical_ (right); thus, we ran one RDA_biogeographical_ for each arthropod group. Each row of the regional matrix presents the locality (L*m*), the plot (P*n*), and the individual plant (A, B, C, D or E_1 to 20_) (1A, right). B) Representation of sampling procedure showing the distribution of twenty plots (30×30 m, grey squares) in the locality *m* (left), as well as the minimum distance between plots (i.e., 50 m). We sampled up to five individual plants per plant species (A, B, C, D, and E) in each plot. At the metacommunity scale (1B, left), we used a species matrix, only plant architectural features as environmental variables, and the distance among *n* plots to perform the RDA_metacommunity_; thus, we ran 12 RDA_metacommunity_ for each arthropod group (see Table 1). See additional details about the definition of biogeographical and metacommunity scales, as well as the analytical procedure in Methods.

In our case study, the challenge is then to understand how these processes assembly communities at different scales (Fig. 1), as we studied very isolated areas that have very similar vegetation type. To investigate how niche- and dispersal-based processes (defined below) affect species composition at metacommunity and biogeographical scales, we studied two vegetation-dwelling arthropod groups along 2,040 km of the Brazilian coast, between -12 and -28 latitude. We selected 12 localities of *restinga* vegetation ranging from Northeast to South of the country (Fig. 1 in S1 Appendix). Whereas biogeographical scale presents the complete pool of localities (Fig. 1A), the metacommunity scale presents the variation occurring between patches within each locality (Fig. 1B). Scarano [22] defined *restinga* vegetation as plant communities that grow in sandy plains (formed in the late Quaternary) occupying stretches between the sea and the Atlantic Rainforest. This vegetation covers about 18,000 km^2^ of the Brazilian coast and the climate ranges from tropical to subtropical [23].

We selected lepidopterans and spiders because both groups are common in this vegetation type; also, those groups have different biology and dispersal capabilities that are important to test our predictions [24]. For instance, lepidopterans are phytophagous and mostly specialized to a single plant family [25]. Thus, plant families with distinct morphology (e.g., leaf size) will affect lepidopteran community composition. Their adults are good dispersers and can fly actively over extensive areas. In addition, ballooning caterpillars (larval phase) can move to a new host plant if the quality of their “old” plant is declining [26]. Spiders, in turn, are generalist predators and most individuals are able to weave webs, which makes habitat structure a noteworthy feature of their life history [27], [28]. Spiders have been considered poor dispersers because they depend on passive movement (ballooning) to reach new localities with suitable conditions [26]. In fact, the dispersal of spiders is considered a high-risk activity, because in cases that spiders land in unfavourable localities, individuals will not be able to reproduce [29] or will die. Thus, spatial structure (geographic distance) probably affect more intensely poor dispersers such as spiders compared to lepidopterans. As a result, the composition of lepidopterans and spiders could be affected by both environmental influence (niche-based process) and distance among suitable habitats (dispersal-based process), although the relative importance of these processes will likely vary between scales and organisms.

We investigated whether the relative importance of niche (plant morphological variables: a proxy of microhabitat variation at the metacommunity scale; and climatic variability at biogeographical scale) and dispersal-based processes (geographical distance: a proxy of dispersal limitation) change at two different spatial grains, i.e., within localities but among different patches (metacommunity scale; Fig. 1B) and among different localities (biogeographical scale; Fig. 1A). We predicted that: (1) dispersal-based processes will affect lepidopterans and spiders at the biogeographical scale, although they will be more important to spiders; (2) niche-based processes will affect lepidopterans and spiders at the metacommunity scale, although they will be more important to lepidopterans; (3) lepidopterans will be less spatially structured in more climatically variable localities, since they are good (active) dispersers [18]; conversely, since spiders are poor (passive) dispersers, they will be spatially structured at small (metacommunity) and large (biogeographical) scales independently of the climate variability; (4) the number of endemic spider species will be higher in climatically stable localities [18].

## Methods

All necessary collect permits were obtained for the described field studies and were licenced by “Instituto Brasileiro do Meio Ambiente e dos Recursos Naturais Renovávies”/IBAMA (proc. n. 14894).

### Study area and sampling

In this study we choose a specific type of *restinga*, called “open *restingas*”, which are characterized by patchy vegetation surrounded by open areas covered either with sand or herbaceous vegetation [30].The main plant families found in open *restingas* belong to the families Arecaceae, Bromeliaceae, Malpighiaceae, Myrtaceae, Rubiaceae and Sapindaceae [30]. Because the selected *restingas* have contrasting climate regimes, we summarized climatic information in Figure S1 and Table S1 in S1 Appendix. We selected 12 localities of *restinga* vegetation along 2,040 km of the Brazilian coast. The average distance between neighboring localities is 199 km (max = 566.3 km, min = 14.7 km), which corresponds to the biogeographical scale (see below).

We considered each plant species as a type of environment (i.e., discrete variable) and we chose each plant species based on their morphology (i.e., values related to plant and leaf size). Specifically, we chose at each locality a bromeliad (family Bromeliaceae), a palm (Arecaceae), and three different dicot plants species with small, medium and large leaves (Tables S2 and S3 in S1 Appendix). These plants occur in natural patches (local community) within each restinga (locality: the metacommunity scale). These five plant species present different morphologies based on canopy and leaf size and shape. In localities without palms (four localities), we substituted them with another common dicot plant with an architecture distinctive from bromeliads and the three other dicots. To standardize across localities, we used differences in plant morphology (e.g., variation in leaf length among plants) to test the effect of local environment on species composition. Differences in plant morphology represent a fine variation in microhabitat structure that affect the demography of species of lepidopterans and spiders [25], [28].

We collected arthropods occurring on five different plant species in each of the 12 localities between September and November of 2009, and June and August of 2010. We selected 20 plots (30×30 m) at least 50 m apart within each locality; we randomized the order of plot sampling. The criterion for choosing these points was the presence of at least three of the five plant species; from each species, we sampled 20 individual plants. Within each plot, we sampled up to 5 individual plants of each species. For example, if we found five bromeliad individuals in each of the four first plots, we did not sample bromeliads in the next plots. This protocol was repeated in each locality and in both years. The plots we chose in the first year were the same in the second year, but new randomizations were performed to decide the order of sampling. To control for the possible effect of different samplings, we used year as a factor in RDA analyses.

We collected arthropods (lepidopterans and spiders) in the branches of each plant using the following protocol: (i) we used 100 L transparent plastic bags to pack four to ten branches (depending on branch size), and cut the branches off; (ii) we carefully shook the bag 20 times to release the arthropods from the branches; (iii) we then removed each branch to check for arthropods in a white tray; (iv) we collected every arthropod visible to the naked eye and conserved them in 75% alcohol. After carefully collecting the arthropods from each branch removed, (v) we weighed (PesolaMedio precision 10 g) all the leaves from these branches to determine total leaf biomass. Thus, even from plants of different sizes, we were able to test whether total leaf biomass affects arthropod abundance. This method was repeated for each individual plant. For bromeliads, however, we did not follow steps i, ii, iii and v; instead, we collected the arthropods (visible to the naked eye) present over the entire plant surface. In addition, we counted the number of leaves of the plant and weighed three leaves (the smallest, one intermediate-sized, and the largest) to estimate total leaf biomass. Then, we multiplied the number of leaves times the average value of the three weighed leaves.

### Definition of scales and proxies of niche- and dispersal based-processes

We considered the whole region from latitudes -12 to -28 (Fig. 1A) as the biogeographical scale (that encompasses three sub-regions of Atlantic Rainforest: [31]). We used 12 values of regional richness along the biogeographical scale. We used macroclimatic and plant morphology as environmental variables at the biogeographical scale. Thus, at the biogeographical scale niche-based processes refer to macroclimatic and plant morphological variables (a proxy of microhabitat variation) and dispersal-based processes refer to the distance among localities. Each region belongs to the same vegetation type (i.e., *restinga*). We defined “regional dispersal” as the dispersal of organisms throughout the biogeographical scale.

The metacommunity scale was defined as the combination of 20 different patches sampled in each locality; these localities are very isolated (i.e., without direct forest connections) from each other and there are several cities (such as São Paulo, Rio de Janeiro and Salvador) and highways that suggest that dispersal among localities is rare or absent. In addition, the average distance among neighboring localities is 195 km. Hereafter we referred to each locality as a metacommunity. We used only plant morphology as microhabitat variables at the metacommunity scale, since the resolution of macroclimatic variables is higher than the distance among plots within each locality. We inferred dispersal-based processes from spatial variables obtained from the distance among patches (plots). Within each locality, we defined the dispersal of organisms among patches as “local dispersal”.

We used the term “niche” to refer to the local (environmental) variables that potentially affect species composition as a result of differential demography of species in different habitat types (species sorting perspective in Metacommunity theory: [12]). Differences in plant morphological characteristics have been considered fundamental predictor of how microhabitat variation affect arthropod composition [27], [28], [32]. Thus, those morphological variable plants are potential niches to be colonized by herbivores (e.g., lepidopterans) and predators (e.g., spiders). If these differences in plant morphology (also called plant architecture) cause contrasting demography among species, as suggested by several studies [28], [32], [33], we argue that plant morphological variables can be used as a proxy of microhabitat variation and thus reflecting niche-based processes. It is important to note that variation in plant morphology can be found at both local and biogeographical scales. In addition, differences in climate variables at the biogeographical scale are also important components of species' niche because they also affect species demography. As a result, we can test whether niche-based processes are operating at different scales with two distinct environmental predictors.

Moreover, Leibold et al. [12] defined the region that supports the metacommunity as the mesoscale [61]. Holt [61] has defined as “the gray zone between the local mechanisms that are the traditional concern of community ecologists and the large-scale processes that are the province of biogeographers and systematics”.

### Environmental variables

At the metacommunity scale, we measured plant morphological variables (micro-habitat variables) such as tree canopy height, plant biomass, the longest and shortest length of tree canopy variables at the plant level, and leaf length, leaf width, distance between the second and third leaf, and the ratio between leaf width and length at the leaf level.

We extracted macroclimatic variables at 1 km^2^ resolution from WorldClim [34]. We used 11 macroclimatic variables related to temperature and precipitation as predictor variables: (1) annual mean temperature, (2) mean diurnal range (max – min temperature), (3) isothermality (mean diurnal range/temperature annual range), (4) temperature seasonality, (5) maximum temperature of the warmest month, (6) minimum temperature of the coldest month, (7) temperature annual range, (8) annual precipitation, (9) precipitation of the wettest month, (10) precipitation of the driest month, and (11) precipitation seasonality (coefficient of variation) [34]. The variables 1, 3 and 8 present annual trends, while variables 2, 3, 4, 7 and 11, and 5, 6, 9, and 10 present seasonality and extreme environmental factors, respectively [34]. Because the distance among plots in the same locality was not large enough to detect differences in macroclimatic variables at a 1 km^2^ resolution, we performed analyses with macroclimatic variables only at the biogeographical scale. To avoid pseudoreplication among macroclimatic variables, we conducted a Principal Component Analysis (PCA) and extracted the first four orthogonal axes (cumulative proportion of 97%) to use as macroclimatic predictor variables. To test the 3 and 4 we performed the PCA just with the variables related to climatic variability (seasonality).

### Statistical analyses

#### Spatial variables

We calculated the range size for each lepidopteran and spider species as the maximum and minimum latitudes (considering the 12 localities) of their occurrence. We attributed the value 1 to the most northeast locality (i.e., latitude -12), value 2 to the second one, and so on. Thus, the most southern locality (i.e., latitude -28) received value 12. For example, the range size of one species that occurs in the whole latitudinal gradient is 11, but the range size of one species that occurs only in one locality is 0. Species with a range size of 0 are thus considered endemics. We are aware that the method used to determine “endemic” species does not guarantee that one species sampled in a specific locality would not be collected in another locality if we had sampled additional habitats. Indeed, by using this method we could not differentiate endemic species from rare species. However, due to sampling limitations, we used the exclusive occurrence of species at one locality as an endemism index [see, e.g.,[35]].

We translated the matrix of plot coordinates (latitude and longitude, Fig. 1A) into spatial predictor variables with spatial eigenvector mapping [36]. Specifically, we used Moran's eigenvector maps (MEMs) [36] based on Gabriel graphs [37], [38] that translate the spatial arrangement of the coordinates into spatial predictors that can be used as explanatory variables in Canonical models [36]. We retained only MEMs with significant values; we also grouped the MEMs as those corresponding to broad (positive autocorrelation) and fine (negative autocorrelation) spatial scales [37]. This technique is suitable for studying the variation of species composition at multiple scales [37]. Thus, we used as spatial predictors in RDA analyses these MEMs presenting broad and fine-scale patterns. We chose MEM to represent spatial predictors because this method is straightforward to study the variation of community composition at multiple scales [37]. S2-2 Figs. and -3 in S2 Appendix show the spatial pattern of those significant spatial components, grouped as broad and fine scale spatial predictors.

#### Tests of the four predictions on diversity structure

We tested predictions 1 (prevalence of dispersal-based processes at the biogeographical scale) and 2 (prevalence of niche-based processes at the metacommunity scale) based on the metacommunity framework by estimating the relative importance of microhabitat variables (plant morphology) and spatial variables (broad and fine-scale MEMs) to arthropod species composition with a Redundancy analysis (RDA) coupled with a Variation Partitioning analysis [39]. The RDA decomposes the total variation in species composition into environmental (*E*) and spatial components (*S*). In addition, we partitioned the total variation into the variance explained exclusively by environmental and spatial variables. We used the unbiased Variance Partitioning method proposed by Peres-Neto et al. [40], which computes the adjusted coefficients of variation for each component. Details of calculation of fractions can be found in Peres-Neto et al. [40] and a comment to recent criticism about variance partitioning method in S2 Appendix. We implemented this analysis for each locality (metacommunity scale analysis: RDA_metacommunity_) and compared all localities (biogeographical scale analysis: RDA_biogeographical_). Prior to RDA_local_ analyses we calculated the variance inflation factor and removed plant morphological variables with values higher than 10 [41]. We added year to the RDA_metacommunity_ models as a factor to control for possible differences of species composition between years. According to prediction 1 (spatial structure), the pure spatial component of the RDA_biogeographical_ will be higher than the pure environmental component for both lepidopterans and spiders, but the relative importance of the pure spatial component will be higher for spiders than for lepidopterans. According to prediction 2, the pure environmental component of the RDA_local_ will be higher than the pure spatial component for both lepidopterans and spiders.

To test whether macroclimate variables affect local environmental and spatial processes, we performed another RDA analysis (RDA_climate_) using the variation explained by each RDA_biogeographical_ fraction (S2 Appendix) against the four scores obtained by the PCA of macroclimate variables. In this analysis it is possible to test whether macroclimate variables at the biogeographical scale predict the variation of each component of arthropod species composition. The RDA_climate_ was done only at the biogeographical scale because at the metacommunity scale the resolution of climate data is not fine enough to show differences among plots. To test whether macroclimate variables explain species richness gradients at the biogeographical scale, we regressed species richness values of each locality against the scores of the PCA analysis obtained from macroclimate variables. We implemented these four analyses (RDA_metacommynity_, RDA_biogeographical_, RDA_climate_ and regression) for lepidopterans and spiders.

To test the prediction 3 (dispersal vs. climatic variability) we used the component S|E (pure spatial) obtained from each arthropod group and regressed it against the scores obtained by the PCA of the macroclimatic variables (S3 Appendix). The higher the values of S|E and PCA scores, respectively, the higher will be the importance of the spatial component (e.g., dispersal limited) and the variability in climate. To test the prediction 4 (endemism vs. environmental stability) we regressed the number of endemic species of each locality against the PCA scores representing the macroclimate variables (S3 Appendix). The lower the value of these variables, the lower is the stability of the environment.

We used R-language environment [42] and the packages ade4, fields, fossil, spacemakeR, rich, and vegan to perform all analyses.

## Results

**Figure 2:**
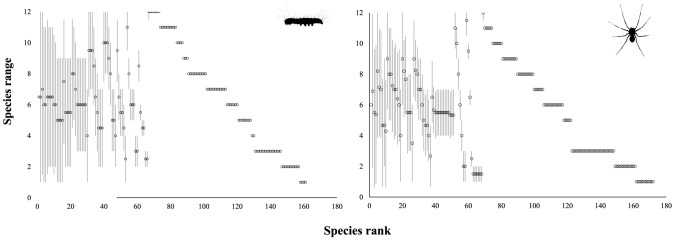
Species' range size of lepidopterans and spiders in relation to their distribution along the Brazilian coast (biogeographical scale). The *X* axis presents the species rank (i.e., species with the greatest range, which occur throughout the whole latitudinal gradient, to species with the smaller range) and the *Y* axis presents species range, i.e., the maximum and minimum occurrences at the latitudinal gradient. Circles present the range centre of each species. Species occurring at one locality (metacommunity) (lowest range) are represented by just a circle.

We collected a total of 333 arthropod species and 1890 individuals in the twelve localities, of which there were 161 species (average richness by locality = 26±8.67 SD) and 766 individuals of lepidopterans (average abundance by locality = 63.8±26.3 SD), and 172 species (27.7±9.87) and 1124 individuals of spiders (93.6±32.8). The range of species along the Brazilian coast was similar between lepidopterans and spiders. Only two species of lepidopterans occurred along the whole latitudinal gradient, and the majority of species (95 for lepidopterans and 104 for spiders) occurred only at one locality (Fig. 2). That is, 59% and 60% of lepidopterans and spider species, respectively, are endemics.

We found that at the biogeographical scale the composition of spiders was mainly explained by broad (R_adj_^2^ = 0.141) and fine spatial scale structures (R_adj_^2^ = 0.016), and the composition of lepidopterans was explained only by the pure spatial structure, i.e., S|E (R_adj_^2^ = 0.061). Indeed, the variance explained by the pure spatial structure were higher for spiders (15.7% summing up broad and fine spatial structures) than lepidopterans (6.1%) (Table 1), as expected in prediction 1. At this scale there is a small, but significant, environmental effect on lepidopterans (R_adj_^2^ = 0.023) and spiders (R_adj_^2^ = 0.011). As expected in prediction 2, at the metacommunity scale the average (i.e., mean value of 12 localities) total variation explaining species composition of lepidopterans was 11.2% (±8.9 SD), of which plant morphological variables (E|S) explained 9.2% of the variation (*P*<0.05 in 8 of 12 localities) and spatial variables (S|E) explained only 0.6% (*P*<0.05 in 3 of 12 localities; Table 1). For spiders, the average (i.e., mean value of 11 localities) total variation explaining species composition was 10.6%, of which 8.3% was explained by plant morphological variables (E|S; *P*<0.05 in 10 of 11 localities) and only 1.5% was explained by spatial variables (S|E; P<0.05 in 6 of 11 localities; Table 1). On average (12 lepidopteran comparisons and 11 spider comparisons), plant morphological variables explained 8.8% (significant in 18 of 23 comparisons) of the variation in those arthropod species composition at the metacommunity scale, while spatial variables explained 1.1% (significant in 9 of 23 comparisons) (see also S4 Appendix). Indeed, the components related to plant morphology and space (S2 Appendix) were not significantly related to bioclimatic variables measured by PCA scores for both lepidopterans (permutation test for RDA: F = 1.302, *P* = 0.34) and spiders (F = 0.763, *P* = 0.67). In summary, spatial structure predominates at the biogeographical scale and microhabitat variables at the metacommunity scale for both lepidopterans and spiders. In addition, species composition did not vary between years for both lepidopterans and spiders (results not shown).

**Table 1:** Explained variation of each component of the partitioning of arthropod species composition (Araneae, Lepidoptera).

	Total	[E ∩ S]	[E]	[S]	[E|S]	[S|E]
**CATTERPILLARS**						
**Biogeographical scale**	0.013	−0.0001	0.001	0.012	0.001	**0.012**
**Metacommunity scale**						
Praia do Forte	0.366	0.017	0.341	0.042	**0.324**	0.025
Salvador	0.074	−0.006	0.080	−0.013	**0.087**	−0.006
Trancoso	0.336	0.039	0.059	0.317	**0.020**	**0.278**
Barra Nova	0.373	0.039	0.280	0.124	**0.249**	**0.093**
Setiba	−0.00004	0.017	0.028	−0.011	0.011	−0.028
Praia das Neves	0.238	0.044	0.097	0.185	**0.053**	**0.141**
Iquipari	0.140	0.023	0.049	0.114	**0.027**	**0.091**
Massambaba	0.009	0.026	0.071	0.041	0.045	0.016
Maricá	0.099	0.035	0.069	0.065	0.035	0.030
Ilha do Cardoso	0.067	0.010	0.092	−0.013	**0.082**	−0.023
Dunas dos Ingleses	0.081	−0.017	0.085	−0.021	**0.102**	−0.003
Dunas de Joaquina	0.037	0.012	0.064	−0.014	0.052	−0.026
**SPIDERS**						
**Biogeographical scale**	0.053	0.001	0.002	0.052	0.001	**0.051**
**Metacommunity scale**						
Praia do Forte	-	-	-	-	-	-
Salvador	0.046	0.012	0.041	0.017	0.029	0.005
Trancoso	0.130	0.013	0.122	0.021	**0.110**	0.008
Barra Nova	0.067	0.002	0.067	0.002	**0.065**	−0.0003
Setiba	0.164	−0.004	0.154	0.007	**0.158**	0.010
Praia das Neves	0.133	0.002	0.125	0.010	**0.123**	0.008
Iquipari	0.110	−0.008	0.088	0.013	**0.097**	**0.022**
Massambaba	0.073	−0.002	0.075	−0.003	**0.077**	−0.002
Maricá	0.193	0.024	0.055	0.162	**0.031**	**0.138**
Ilha do Cardoso	0.027	0.003	0.021	0.009	0.018	0.006
Dunas dos Ingleses	0.160	0.023	0.146	0.037	**0.123**	0.014
Dunas de Joaquina	0.134	−0.004	0.126	0.005	**0.130**	0.008

[E] and [S] represent the environmental and spatial components without control for the autocorrelation. [E|S] represents pure environmental (plant morphology) effects. [S|E] represents pure spatial effects. The spatial variation presenting broad and fine scale spatial variation was significant only for spiders. Bold values indicate significant values (*P*<0.05) of each pure fraction. For Praia do Forte (only spiders) we do not have enough data to perform variance partitioning.

The spatial component (a proxy of dispersal limitation) associated with lepidopteran species composition was negatively related to climatic variability (*F* = 18.41, *P* = 0.002 for PCA3; Tables 2 and 3), as expected in prediction 3. The PCA3 axis was positively related to precipitation seasonality and negatively related to mean temperature diurnal range. The number of endemic spider species was significantly associated with PCA1 (F = 6.57, *P* = 0.037, Tables 2 and 3); PCA1 was positively related to temperature seasonality and annual range and negatively related to isothermality (Table 3). Therefore, spider endemism was higher at localities with lower isothermality (i.e., lower temperature diurnal range compared to annual temperature range:[34]) and lower temperature seasonality, as expected in prediction 4. The number of endemic lepidopterans was not associated with PCA1 (F = 1.69, *P* = 0.246, Table 2). The PCA2 axis was negatively correlated to mean temperature diurnal range, maximum temperature of the warmest month, and precipitation of the wettest month (Table 3 and Table S3-1 in S3 Appendix).

**Table 2:** Results of the linear regression used to test the effect of bioclimatic variables (i.e., scores of the PCA analysis) on pure spatial components (a proxy of dispersal limitation) and endemism of lepidopterans and spiders.

**Lepidopterans**				
*Pure spatial component [S|E]*	*F*		*P*	
PCA1	1.505		0.259	
PCA2	0.02		0.895	
PCA3	7.54		**0.029**	
*Endemic species*				
PCA1	3.39		0.108	
PCA2	0.52		0.495	
PCA3	0.19		0.916	
**Spiders**				
*Pure spatial component [S|E]*	*F*		*P*	
PCA1	6.69		**0.036**	
PCA2	1.89		0.211	
PCA3	0.05		0.822	
*Endemic species*				
PCA1	6.57		0.037	
PCA2	0.74		0.417	
PCA3	0.34		0.576	

**Table 3:** PCA loadings of variables of climate seasonality associated with the PCA axis.

Seasonality	PCA1	PCA2	PCA3	PCA4
Mean diurnal range	0.224	0.703	0.492	−0.163
Isothermality	−0.528	0.372	0.152	0.709
Temperature seasonality	0.573	−0.213	−0.072	0.685
Temperature annual range	0.574	0.174	0.183	0.036
Precipitation seasonality	0.113	0.539	−0.834	−0.018

Additional information about bioclimatic variables and PCA analysis in S3 Appendix.

## Discussion

The growing evidence that neither dispersal-based nor niche-based processes exclusively explain the patterns of similarity among communities [43]–[45] illustrates that these processes operate successively and simultaneously to assemble communities [17], [46]. We found that, at the biogeographical scale, mainly the geographical distance explained the variation in species composition of lepidopterans and spiders suggesting that dispersal-based processes control large scale diversity patterns. Conversely, we showed that plant morphological variables (a proxy of microhabitat variation) explained most of the variation in the species composition of lepidopterans and spiders at the metacommunity scale, which reinforces that niche-based processes are pervasive in determining metacommunity scale patterns. Thus, although the regional species pool throughout the biogeographical scale can influence metacommunities by providing propagules [20], differences in microhabitat preferences among species (or other selection factors) will probably determine the eventual local distribution of species.

As we expected from prediction 1, lepidopteran and spider communities were spatially structured at the biogeographical scale, which suggests that geographic distance is constraining the distribution of these terrestrial arthropods. Under dispersal limitation at the biogeographical scale, this spatial pattern may arise from changes in species abundance throughout the evolutionary history of these arthropod communities along different regions, thus a combination of interactions between large scale dispersal limitation events, speciation, and stochastic events [47], [48]. For example, we found two spider species from the genus *Psecas* (Salticidae) occurring only on bromeliads, while *Psecas* sp1 occurs from latitude -18 to -21, *Psecas* sp2 occurs from latitude -25 to -27. The distinct spatial distribution of these two species coincides with the divergent distribution of endemic anurans from the genus *Rhinella*, in which genetic breaks in their phylogeny were spatially concordant with geographic barriers (e.g., rivers) in the Atlantic Forest [49]. These barriers could limit dispersal and therefore isolate species in different metacommunities, reduce gene flow and increase allopatric speciation [18]. Thus, allopatric speciation may interact with dispersal to generate and maintain regional species pool across the biogeographical scale.

**Figure 3:**
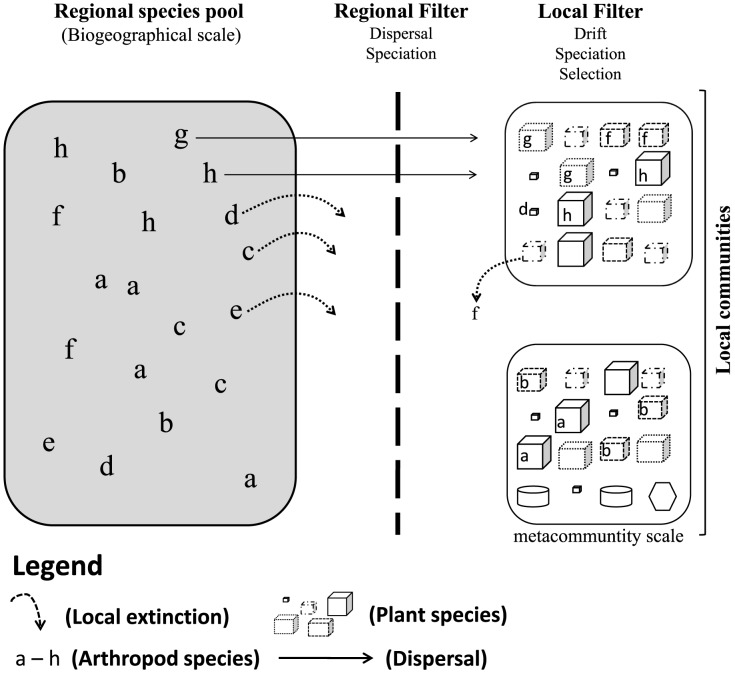
Schematic representation of the proposed hierarchical assembly of lepidopteran and spider species composition (represented by letters a to h). The composition of metacommunities will be a balance of species from the biogeographical species pool that are able to disperse to each metacommunity (solid arrows). Some species are not able to colonize metacommunities (black dotted arrows). Throughout the time dispersal and allopatric speciation will affect both the biogeographical species pool and thus metacommunities. Within each local community, the selection of arthropod of plant species with specific morphologies (presented as different shapes) will also determine species composition. In addition, ecological drift, speciation and local extinction (grey dotted arrow) could eliminate species from metacommunities even when species' “preferred” conditions (such as specific plant morphology, a microhabitat variation) are found.

As expected in prediction 2, we showed that at the metacommunity scale plant morphological variables explain the variation in species composition of lepidopterans and spiders. Microhabitat variation (i.e., leaf width and canopy height) affected local lepidopteran and spider communities at different latitudes. This result adds voice to several studies claiming that plant species composition or plant morphology (clearly a fine variation in environmental characteristics), has pivotal importance in assembling local arthropod communities [28], [32], [33], [50]. It has been suggested that locally, plant species and their morphological variation are more important than climate variables [51]. This does not mean, however, that the mechanisms affecting the composition of lepidopterans and spiders are the same (e.g., plant phylogenetic relatedness has been considered important for lepidopterans [25] and plant morphology for spiders [28]), though. We argue that processes such as dispersal and speciation (typically occurring at the biogeographical scale) act together with selection (e.g., typically local processes such as habitat preferences, which occur at the metacommunity scale) and speciation in determining the composition of communities (Fig. 3) [48], [52]. In addition, these results also indicate that dispersal-based processes determine how much of the regional pool will occur locally [52], but then other processes (selection, speciation) will act at the metacommunity scale. We have shown that ∼91% of the variation in species composition at the metacommunity scale was unexplained, even after taking into account spatial and environmental variation (microhabitat variables). Even though shading gradient for spiders [53] and plant secondary chemical components for lepidopterans [54] are known drivers of their community composition, we did not include them because of experimental design restrictions. Nevertheless, the amount of variation explained by the chosen parameters is in the common range [55], [56], and other studies have shown that plant species composition and their morphological variables are the main/sufficient drivers of community composition [51], [57]. We speculate that the unexplained variation could also be attributed to neutral processes (via ecological drift) [58] acting at the metacommunity scale along with niche-based processes, varying in relevance as a matter of scale. As a result, each local arthropod metacommunity could be organized by microhabitat variation, but the relative importance of this variation depends on capability of species (from the regional pool along the biogeographical scale) to colonize each locality. Thus, the presence of certain species in the region does not mean that organisms of this species will necessarily disperse to all local metacommunities and find their preferred habitats, which may explain the 91% of unexplained variation in species composition. For example, the bromeliad-living spider *Psecas* sp. did not occur at the Trancoso's *restinga* (Fig. 1) although its microhabitat (bromeliad) is densely distributed in this locality. Taken together, high spatial structure at the biogeographical scale, local determinism associated with microhabitat variation and the remaining 91% of unexplained variation illustrate that regional and local processes are not mutually exclusive [13] and probably interact to assemble metacommunities.

Differences in dispersal capabilities among organisms can also affect both the species available along the latitudinal gradient and the response to climate variability. For example, lepidopterans were less spatially structured in localities with more variability in mean temperature diurnal range. This result suggests that species need to disperse longer extensions to find suitable patches, as expected for good dispersers such as lepidopterans (prediction 3). On the other hand, the number of endemic spiders was higher in climatically stable localities (i.e., lower isothermality and precipitation seasonality), as expected in prediction 4. We suggest that differences in dispersal between adult lepidopterans and spiders may explain the differential effects of climate on their spatial structure and endemism. On the one hand, lepidopterans (adults) are dispersers that actively choose the locality and the host plant to oviposit, resulting in a “deterministic” occurrence. These adults may occur, for example, in localities with a specific range of temperature [59] through direct active choice. In fact, in localities with more instability in temperature, lepidopterans were more dispersal-limited. On the other hand, the majority of spiders disperse passively using silk threads, resulting in a “stochastic” occurrence. Thus, in localities with suitable climatic conditions there will be more species of lepidopterans because these organisms can actively choose the best quality localities. However, in those suitable localities we can find more endemic spiders because in localities with unsuitable conditions (e.g., unstable climate) few spider species will survive. These results highlight the importance of considering differences in dispersal abilities among species to obtain a more predictive metacommunity model to explain large-scale patterns, such as the latitudinal gradient [18].

By integrating processes that operate at different scales [60], we suggested that dispersal processes at the biogeographical scale, coupled with plant morphological variables (microhabitat variation) at the metacommunity scale are interactively affecting small and large scale diversity patterns. Our results suggest that biogeographical and evolutionary processes (mainly dispersal and speciation) are operating in assembling species composition at large scales, but niched-based processes are acting within different metacommunities throughout the region in driving small-scale diversity patterns. More importantly, these processes are acting successively and simultaneously to assemble communities. This result is a complementary vision of previous studies on arthropod biogeography done at the temperate zone [20], [60], because it opens an unanswered question of why tropical arthropods have more spatial and less environmental structure in the biogeographic scale than temperate organisms [20]. Also, these findings help to reconcile two separate scientific fields (metacommunity and biogeography), which suggest that future work can then build on this approach to explicitly integrate the evolutionary history of organisms to explore, for example, the evolutionary origin of regional species pools.

## Supporting Information

S1 Appendix **Information of macroclimate variables, plant species and families at the 12 sampled sites.**
(PDF)

S2 Appendix **Auxiliary description of methods and statistical analyses.**
(PDF)

S3 Appendix **Macroclimatic variables used and details of the Principal Components Analysis (PCA) used to control for autocorrelation of those variables.**
(PDF)

S4 Appendix **F values of the Redundancy analysis (RDA) between species composition and plant traits.**
(PDF)
