# Supplementary material for: Metacommunity versus Biogeography: A Case Study of Two Groups of Neotropical Vegetation-Dwelling Arthropods
Source: PLoS One. 2014 Dec 30;9(12):e115137. doi: 10.1371/journal.pone.0115137 (PMC4280172; doi:10.1371/journal.pone.0115137)
Supplement: S1 Appendix — Information of macroclimate variables, plant species and families at the 12 sampled sites. (PDF) [file pone.0115137.s001.pdf]

Appendix S1: Information of macroclimate variables, plant species and families at the 12 sampled sites.

We conducted this study on 12 *restinga* sites along the Brazilian coast (Fig. 1, Manuscript), ranging from South to Northeast of the continent (Table S1). We used the macroclimate variables obtained from Hijmans et al. (2005). They defined the macroclimate variables as bioclimatic 1 to 19, but we used bio1 (annual mean temperature), bio2 (monthly mean value of maximum – minimum temperature), bio3 (isothermality, which is the ratio between bio2 and bio7), bio4 (temperature seasonality), bio5 (maximum temperature of the warmest month), bio6 (minimum temperature of the coldest month), bio 7 (temperature annual range), bio 12 (annual precipitation), bio 13 (precipitation of the wettest month), bio 14 (precipitation of the driest month), bio 15 (precipitation seasonality) (Hijmans et al. 2005; http://www.worldclim.org/bioclim). These bioclimatic variables are derived from monthly values of temperature and precipitation with 2.5 arc-minutes resolution.

To show the geographic and climatic dendrograms representing the distance among sites, we implemented a Hierarchical Cluster analysis (Figure S1). These clusterings were based in an Euclidian distance matrix for climatic variables and in a Great Circle distance for geographic distance among sites. We used R-language environment (R Development Core Team 2011) and the packages fields and stats.

We summarized the information about plant architecture sampled at each site and the morphospecies in the Table S2 and S3.

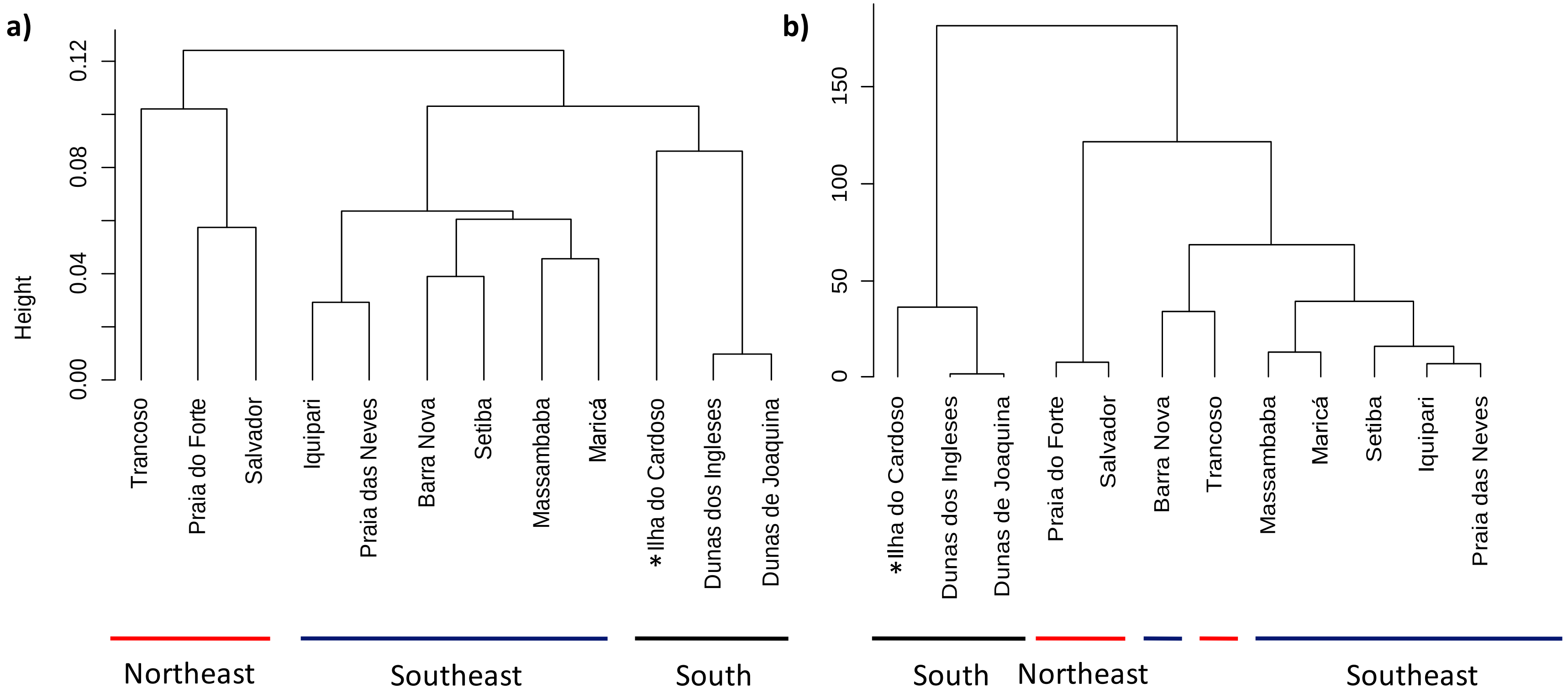

Figure S1. UPGMA dendrograms based on (a) climatic and (b) geographic distance among the 12 sites. Mantel statistics (*r*) of the correlation between the distance matrices was 0.575 (*P* = 0.001). (*) In this analysis, "Ilha do Cardoso" was clustered in the south

group, but considering the Brazilian regions, it is from the Southeast region. However, this site is localized in the most southern city from this region.

Table S1-1. Geographic coordinates (Longitude and Latitude) and bioclimatic variables of each site obtained from Hijmans et al. (2005) method. The code of the bioclimatic variables (bio1-7, bio12-15) is the same suggested by the authors of the original description (Hijmans et al. 2005). See text to detailed information about bioclimatic variables.

**Site**	Longitude	Latitude	bio1	bio2	bio3	bio4	bio5	bio6	bio7	bio12	bio13	bio14	bio15
Praia do Forte	-38.0023	-12.5694	24.66	7.41	66.74	131.41	30.3	19.2	11.1	1749	312	73	51.61
Salvador	-38.3228	-12.9194	24.99	6.44	65.73	117.59	30	20.2	9.8	1275	193	50	45.73
Trancoso	-39.1003	-16.6556	24.46	7.63	65.80	133.62	30.1	18.5	11.6	1477	155	91	18.33
Barra Nova	-39.7387	-18.9573	24.10	8.54	62.35	166.30	31	17.3	13.7	1279	180	55	41.96
Setiba	-40.4164	-20.6054	24.56	7.80	58.65	177.06	31.5	18.2	13.3	1100	172	47	44.71
Praia das Neves	-40.9758	-21.2355	23.33	8.48	59.74	185.23	29.9	15.7	14.2	969	167	27	53.06
Iquipari	-41.0321	-21.7285	23.05	8.55	58.97	189.76	30	15.5	14.5	1022	148	28	46.69
Massambaba	-42.1289	-22.9449	23.03	6.48	56.30	180.86	28.9	17.4	11.5	870	114	39	32.06
Marica	-42.8479	-22.9608	23.35	7.96	55.27	214.49	30.6	16.2	14.4	1182	147	51	36.86
Ilha do Cardoso	-47.9136	-25.0673	22.63	8.60	50.29	297.60	31	13.9	17.1	2469	374	84	46.74
Dunas dos Ingleses	-48.4040	-27.4945	20.04	7.21	47.42	278.83	28	12.8	15.2	1521	198	74	34.13
Dunas de Joaquina	-48.4588	-27.6192	19.65	6.86	45.72	282.57	27.5	12.5	15	1481	203	74	35.61

Table S1-2. Plant species (or morphospecies) selected in each site. The number of plant species varies from 5 to 6. Asterisks denote site that we did not find palm species; thus, we substituted for another dicot species with architecture distinctive from the other dicots.

**Site**	Bromeliad 1	Bromeliad 2	Palm	Small-leaved dicot	Medium-leaved dicot	Large-leaved dicot
Praia do Forte	*Aechmea* cf. *aquilega*	-	*Allagoptera* sp.	Myrtaceae sp.1	Rubiaceae sp.	*Manilkara* sp.
Salvador	*Hohenbergia litorallis*	-	*Allagoptera* sp.	*Byrsonima* sp.	Anarcadiaceae sp.	Sapotaceae sp.
Trancoso	*Aechmea blanchetiana*	*Bromelia antiacantha*	-	Humiriaceae sp.	*Matayba discolor*	*Anacardium* sp.
Barra Nova	Bromeliaceae sp.1	*Quesnelia arvensis*	*Allagoptera* sp.	*Eugenia* sp.	*Protium* sp.	*Clusia* sp.
Setiba	*Aechmea lingulata*	*Vriesea procera*	*Allagoptera* sp.	*Baccharis* sp.	Myrtaceae sp.2	*Clusia* sp.
Praia das Neves	*Aechmea lingulata*	*Vriesea procera*	*Allagoptera* sp.	unindentified	*Byrsonima* sp.	*Clusia* sp.
Iquipari	*Neoregelia cruenta*	*Aechmea nudicaulis*	*Allagoptera* sp.	*Eugenia* sp.	*Pera* sp.	*Clusia* cf. *hilariana*
Massambaba	*Neoregelia cruenta*	-	*Allagoptera* sp.	*Pithecellobium* sp.	*Byrsonima* sp.	*Clusia* sp.
Marica	*Neoregelia cruenta*	-	*Allagoptera* sp.	*Pithecellobium* sp.	*Byrsonima* sp.	*Clusia fluminensis*
Ilha do Cardoso	*Quesnelia arvensis*	-	*Tibouchina clavatium**	Asteraceae sp.1	*Dodonaeae viscosa*	*Dalbergia* sp.
Dunas dos Ingleses	*Aechmea* sp.	Bromeliaceae sp.2	Myrtaceae sp.3*	Asteraceae sp.1	*Dodonaeae viscosa*	*Clusia criuva*
Dunas de Joaquina	*Aechmea* sp.	Bromeliaceae sp.2	Myrtaceae sp.3*	Asteraceae sp.1	*Dodonaeae viscosa*	*Clusia criuva*

Table S1-3. Plant species (or morphospecies) and their families.

Species (or morphospecies)	Plant family
Aechmea lingulata	Bromeliaceae
Aechmea nudicaulis	Bromeliaceae
Aechmea sp.	Bromeliaceae
Allagoptera sp.	Arecaceae
Byrsonima sp.	Malpiguiaceae
Clusia cf. *hilariana*	Clusiaceae
Clusia criuva	Clusiaceae
Clusia fluminensis	Clusiaceae
Clusia sp.	Clusiaceae
Dalbergia sp.	Fabaceae
Dodonaeae viscosa	Sapindaceae
Eugenia sp.	Myrtaceae
Neoregelia cruenta	Bromeliaceae
Pera sp.	Euphorbiaceae
Pithecellobium sp.	Fabaceae
Protium sp.	Burseraceae
Quesnelia arvensis	Bromeliaceae
Tibouchina clavatium	Melastomataceae
Vriesea procera	Bromeliaceae

Literature cited

Hijmans, R.J., Cameron, S.E., Parra, J.L., Jones, P.G. & Jarvis, A. (2005) Very high resolution interpolated climate surfaces for global land areas. *International Journal of Climatology*, **25**, 1965–1978.

R Development Core Team (2011) *R: A language and environment for statistical computing*. R Foundation for Statistical Computing, Vienna, Austria. http://www.R-project.org.
