# Supplementary material for: Metacommunity versus Biogeography: A Case Study of Two Groups of Neotropical Vegetation-Dwelling Arthropods
Source: PLoS One. 2014 Dec 30;9(12):e115137. doi: 10.1371/journal.pone.0115137 (PMC4280172; doi:10.1371/journal.pone.0115137)
Supplement: S2 Appendix — Auxiliary description of methods and statistical analyses. (PDF) [file pone.0115137.s002.pdf]

Appendix S2. Auxiliary description of methods and statistical analyses

1. Spatial patterns and MEM variables

The significant MEM variables presenting broad and fine scale patterns were plotted as a function of each replicate. Thus, patches within localities are plotted in *y* axis and significant MEMs (broad and fine scale) in *x* axis (see details in main text).

Fig. S2 – 1. Broad scale spatial predictors of spider metacommunities. The *y* axis (index) presents the raw data (replicates – patches – within each locality) of the matrix of coordinates. The values range from south (left: index = 1) to northeast (right: index=140).

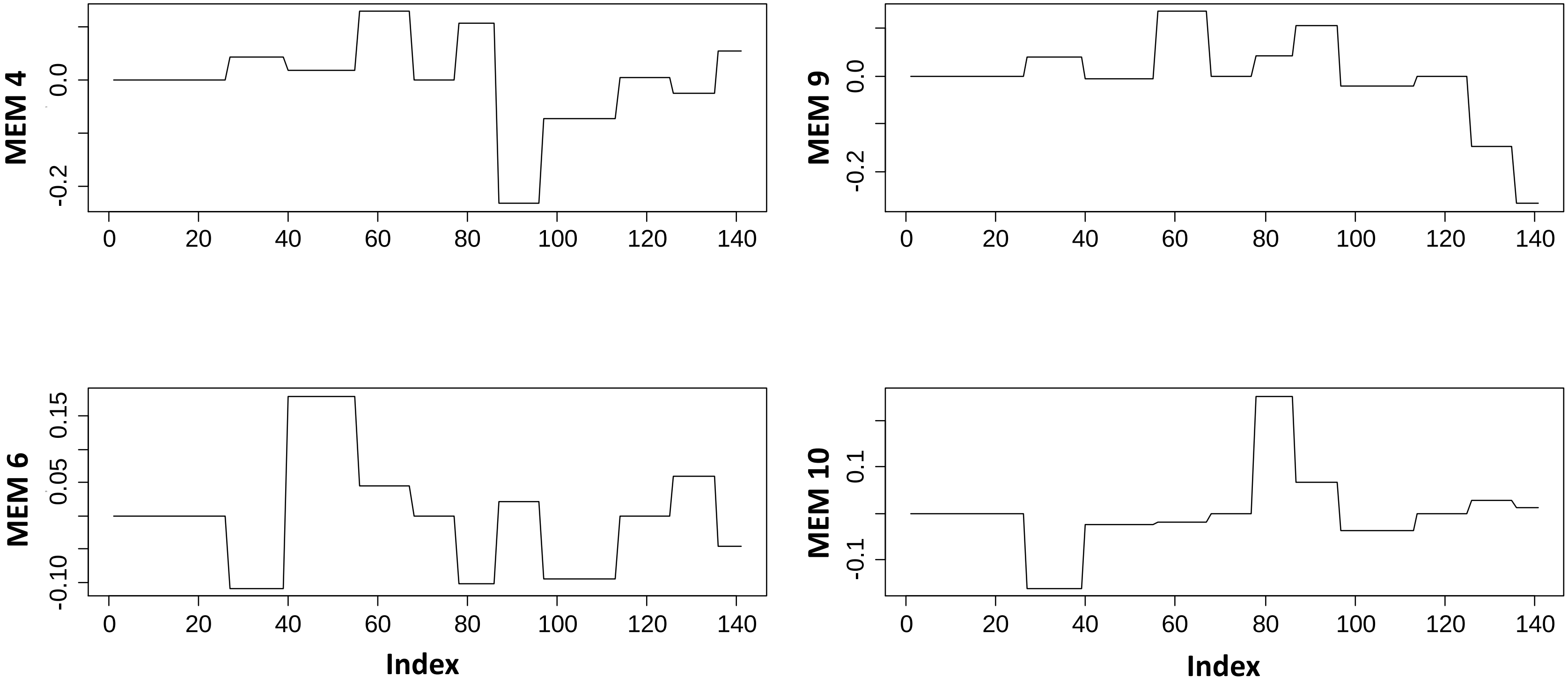

Fig. S2 – 1. Broad scale spatial predictors of spider metacommunities. The *y* axis (index) presents the raw data (replicates – patches – within each locality) of the matrix of coordinates. The values range from south (left: index = 1) to northeast (right: index=140).

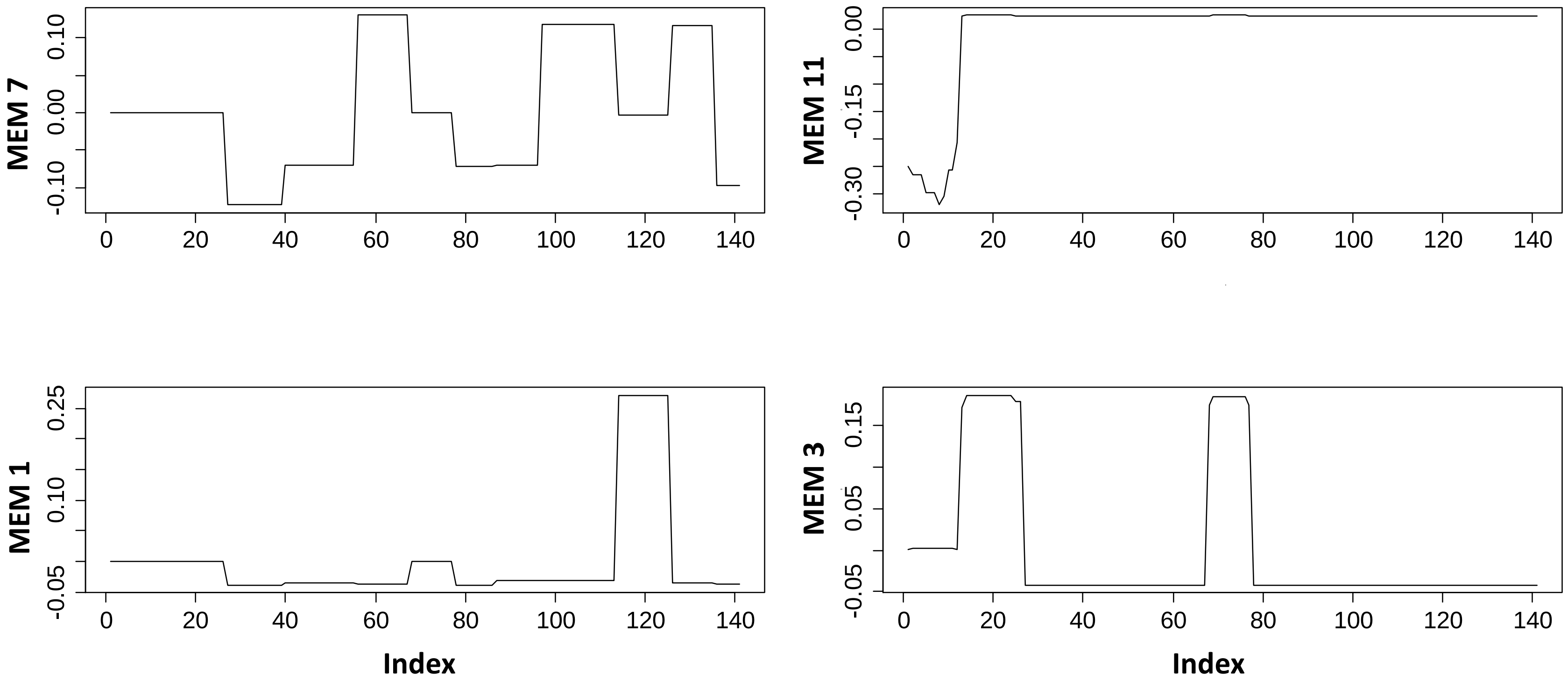

Fig. S2 – 2. Broad scale spatial predictors of spider metacommunities. The *y* axis (index) presents the raw data (replicates – patches – within each locality) of the matrix of coordinates. The values range from south (left: index = 1) to northeast (right: index=140).

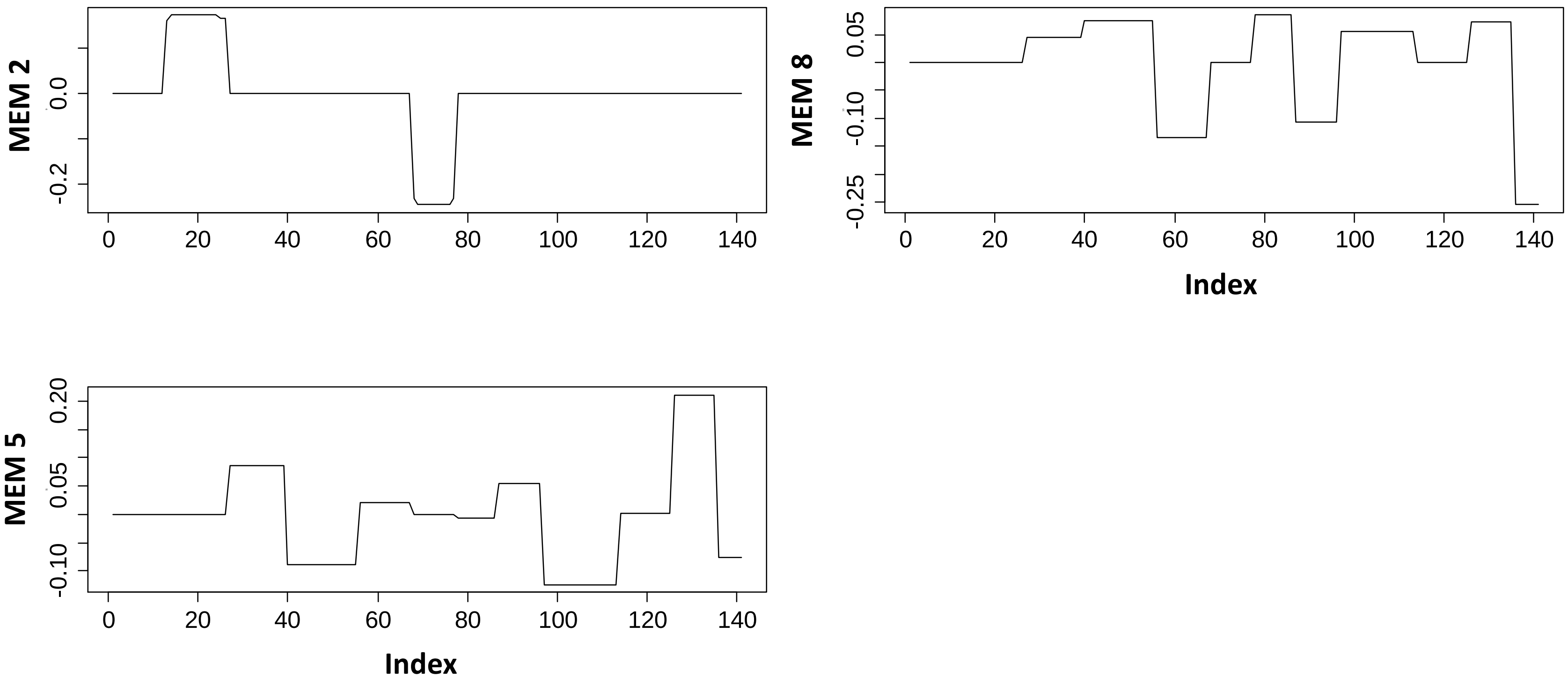

Fig. S2 – 3. Broad scale spatial predictors of lepidopteran metacommunities. The *y* axis (index) presents the raw data (replicates – patches – within each locality) of the matrix of coordinates. The values range from south (left: index = 1) to northeast (right: index=140).

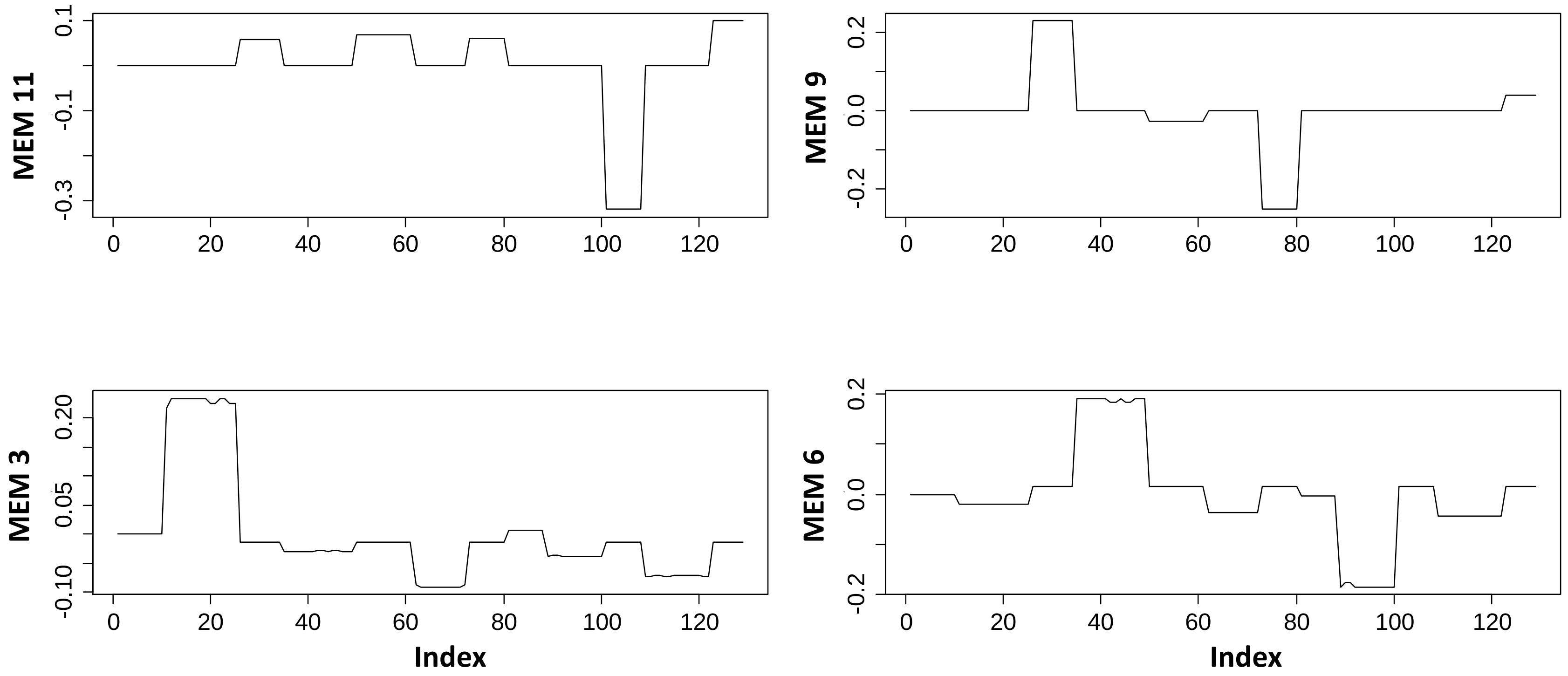

Fig. S2 – 3. Broad scale spatial predictors of lepidopteran metacommunities. The *y* axis (index) presents the raw data (replicates – patches – within each locality) of the matrix of coordinates. The values range from south (left: index = 1) to northeast (right: index=140).

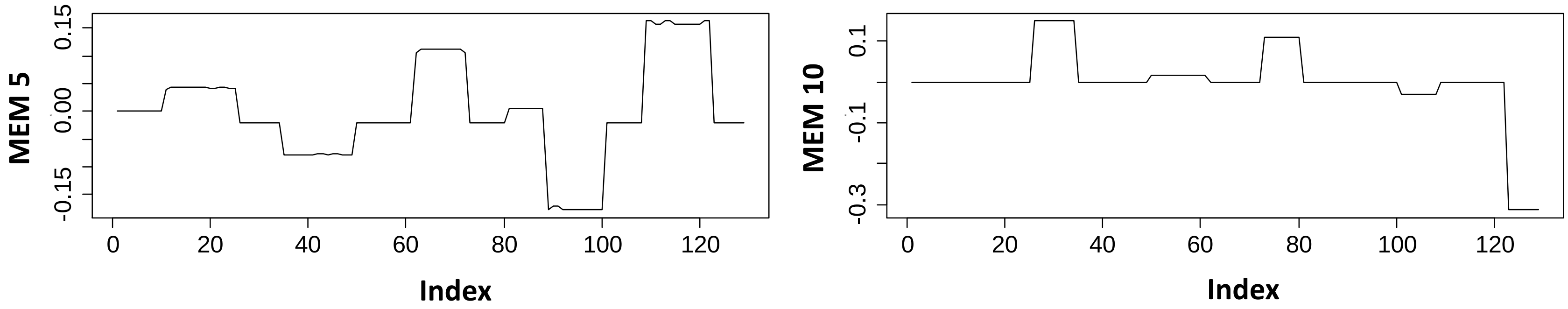

2. Fractions of the variance partitioning and problems with this method

The fractions obtained with the partitioning method were: total explained variation (E+S), environmental (E) and spatial (S) variations. In addition, we obtained pure environmental without the spatial component (E|S), pure spatial without the environmental component (S|E), the shared explanation of environmental and spatial components (S∩E), and the residual variation (Peres-Neto *et al.*, 2006). The variance explained by environmental and spatial (broad and fine) components are plotted below in figures S3-4 and -5.

Despite recent criticism about the accuracy of variance partitioning in disentangling the influence of multiple components in species composition (Gilbert & Bennett, 2010), we believe that our sampling design diminished the problems indicated by these authors. For example, they showed that sampling configuration (e.g., contiguous plots vs. distant plots) affects the estimation of the spatial component (S), and that the linear terms for environmental variables are insufficient to be tested against the complexity of spatial models. We argue that at the metacommunity scale the distance among plots is not dispersal limiting (in fact, the spatial component was not significant; see Results), and at the biogeographical scale, distance among localities is large enough to be detected by the partitioning method (see Results). In addition, by using pre-determined plant traits, the environmental variables (plant traits) were not spatially autocorrelated (see Results). In a recent work, Diniz-Filho *et al*. (2012) showed that the percentage of explained variation of the pure spatial component [c] decreases with the number of non-neutral species in the community. At the latitudinal scale, we showed that the explanation of the pure spatial component was not most important for the composition of arthropods, suggesting that there is indeed a strong local drift of many neutral species associated with each plant trait component.

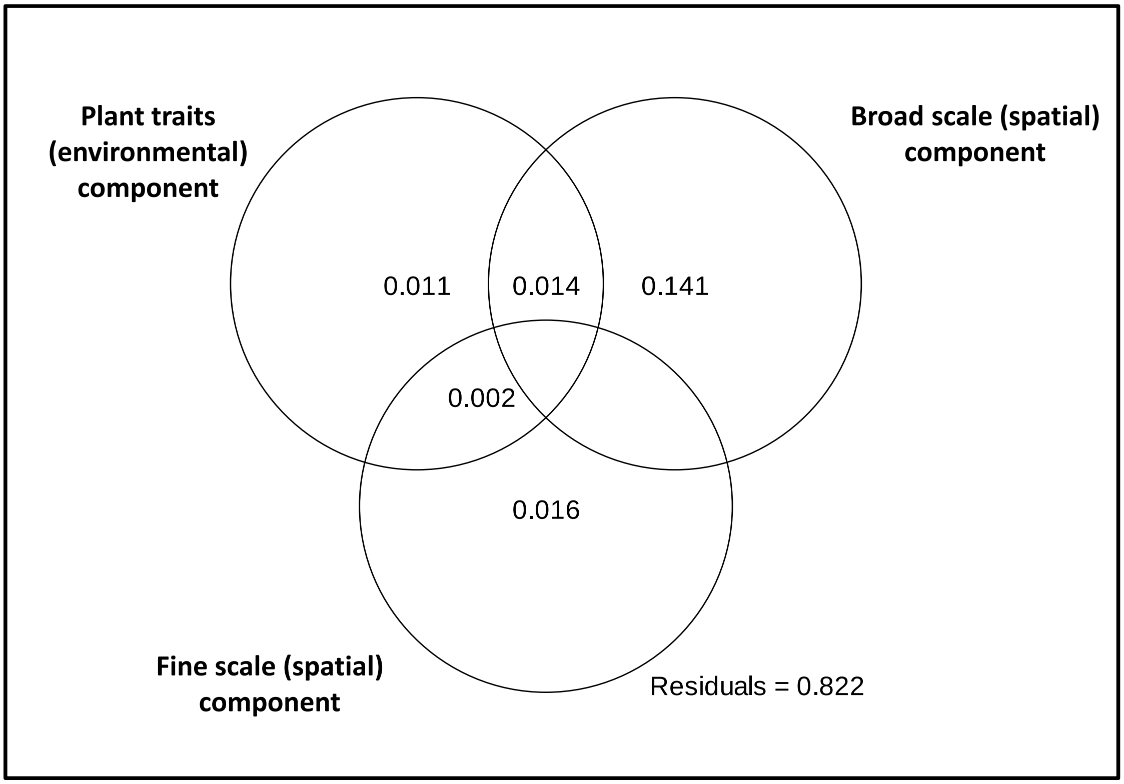

Figure S2-4. Variance partitioning of spider communities (results and *P* values provided in the main text).

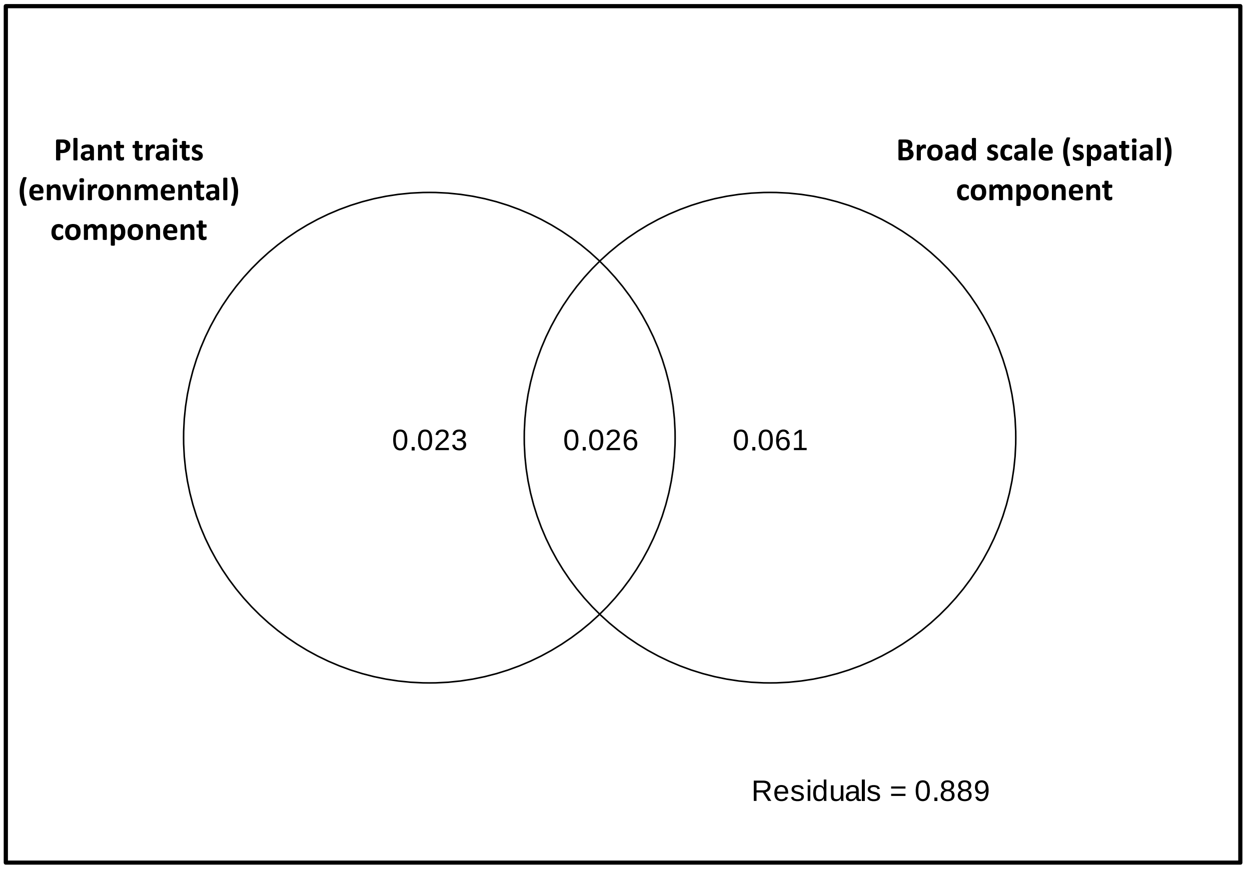

Figure S2-5. Variance partitioning of lepidopteran communities (results and *P* values provided in the main text).

References

Borcard, D. & Legendre, P. (2002) All-scale spatial analysis of ecological data by means of principal coordinates of neighbour matrices. *Ecological Modelling*, **153**, 51-68.

Borcard, D., Gillet, F. & Legendre, P. (2011) *Numerical Ecology with R*. Springer, New York.

Diniz-Filho, J.A.F., Siqueira, T., Padial, A.A., Rangel, T.F., Landeiro, V.L. & Bini, L.M. (2012) Spatial autocorrelation analysis allows disentangling the balance between neutral and niche processes in metacommunities. *Oikos*, **121**, 201–210.

Dray, S., Legendre, P. & Peres-Neto, P.R. (2006). Spatial modelling: a comprehensive framework for principal coordinate analysis of neighbour matrices (PCNM). *Ecological Modelling*, **196**, 483-493.

Gilbert, B. & Bennett, J.R. (2010) Partitioning variation in ecological communities: do the numbers add up? *Journal of Applied Ecology*, **47**, 1071–1082.

Peres-Neto, P.R., Legendre, P., Dray, S. & Borcard, D. (2006) Variation partitioning of species data matrices: Estimation and comparison of fractions. *Ecology*, **87**, 2614-2625.
