# Supplementary material for: Metacommunity versus Biogeography: A Case Study of Two Groups of Neotropical Vegetation-Dwelling Arthropods
Source: PLoS One. 2014 Dec 30;9(12):e115137. doi: 10.1371/journal.pone.0115137 (PMC4280172; doi:10.1371/journal.pone.0115137)
Supplement: S3 Appendix — Macroclimatic variables used and details of the Principal Components Analysis (PCA) used to control for autocorrelation of those variables. (PDF) [file pone.0115137.s003.pdf]

Appendix S3: Macroclimatic variables used and details of the Principal Components Analysis (PCA) used to control for autocorrelation of those variables.

We used 11 macroclimatic variables related to temperature and precipitation as predictor variables: (1) annual mean temperature, (2) mean diurnal range (max – min temperature), (3) isothermality (mean diurnal range/temperature annual range), (4) temperature seasonality, (5) maximum temperature of the warmest month, (6) minimum temperature of the coldest month, (7) temperature annual range, (8) annual precipitation, (9) precipitation of the wettest month, (10) precipitation of the driest month, and (11) precipitation seasonality (coefficient of variation) (Hijmans *et al.*, 2005). The variables 1, 3 and 8 present annual trends, while variables 2, 3, 4, 7 and 11, and 5, 6, 9, and 10 present seasonality and extreme environmental factors, respectively (Hijmans *et al.*, 2005). Because the distance among plots in the same locality was not large enough to detect differences in macroclimatic variables at a 1 km^2 resolution, we performed analyses with macroclimatic variables only at the regional scale. Once these bioclimatic variables were strongly autocorrelated, we performed a Principal Component Analysis (PCA) and extracted the first four orthogonal axes (cumulative proportion of 97%) to use as macroclimatic predictor variables. To test the predictions of Jocqué *et al.* (2010) we performed the PCA just with the variables related to climatic variability (seasonality).

Table S3-1. Component loadings of 11 macroclimatic variables on the first 4 components that accounted for 97% of the total variance. Macroclimatic variables accounting for most variability in each principal component are shown in bold.

	**PC1 (50.5%)**	**PC2 (23.6%)**	**PC3 (14.8%)**	**PC4 (8.5%)**
**bio1**	**-0.828**	-0.488	0.136	-0.008
**bio2**	0.157	**-0.756**	-0.285	**0.492**
**bio3**	**-0.897**	-0.264	0.152	0.022
**bio4**	**0.959**	0.027	-0.097	-0.004
**bio5**	-0.406	**-0.837**	0.039	0.140
**bio6**	**-0.918**	-0.152	0.235	-0.146
**bio7**	**0.845**	-0.325	-0.256	0.258
**bio8**	**0.710**	-0.485	**0.441**	-0.120
**bio9**	0.638	-0.580	0.235	-0.383
**bio10**	0.478	-0.038	**0.834**	0.006
**bio11**	0.046	-0.439	**-0.596**	**-0.621**
