# Supplementary material for: Metacommunity versus Biogeography: A Case Study of Two Groups of Neotropical Vegetation-Dwelling Arthropods
Source: PLoS One. 2014 Dec 30;9(12):e115137. doi: 10.1371/journal.pone.0115137 (PMC4280172; doi:10.1371/journal.pone.0115137)
Supplement: S4 Appendix — F values of the Redundancy analysis (RDA) between species composition and plant traits. (PDF) [file pone.0115137.s004.pdf]

**Appendix S4.**

**Table S1**. *F* values of the Redundancy analysis (RDA) between species composition and plant traits. To test for the significance of each plant trait we implement 1000 randomizations. We considered tree canopy height (CH), plant biomass (PB), the longer (LLTC) and shorter (SLTC) length of tree canopy as plant level variables, and leaf length (LL), leaf width (LW), distance between the second and third leaf (DBL), and the ratio between leaf width and length (RLL) as leaf level variables. Values of *F* in red mean significant *P* values ($P < 0.05$). The sites were organized in the table from the Northeastern (Praia do Forte or Salvador) to the Southern (Dunas de Joaquina). See Fig. 1 (Manuscript) for additional geographical details.

| **Organisms**/sites | **Plant level** | | | | **Leaf level** | | | | **Year** |
|---|---|---|---|---|---|---|---|---|---|
| | CH | LLTC | SLTC | PB | LL | LW | HBL | RLL | |
| **Catterpillars** | | | | | | | | | |
| Praia do Forte | 6.098 | 5.887 | - | 0.585 | - | 4.374 | 1.175 | 2.585 | 1.399 |
| Salvador | 2.631 | 1.291 | 1.084 | 1.503 | - | 1.717 | 0.674 | 1.289 | 1.53 |
| Trancoso | 1.818 | 0.985 | 1.139 | 1.494 | 1.219 | 3.694 | 0.925 | 2.423 | 4.806 |
| Barra Nova | 3.888 | 5.529 | - | 0.610 | 1.565 | 1.479 | 1.258 | 1.518 | 1.292 |
| Setiba | 1.652 | 0.987 | 1.235 | 1.166 | 1.285 | - | 0.896 | 1.561 | 1.348 |
| Praia das Neves | 1.775 | 1.968 | 0.714 | 1.041 | 2.164 | 2.496 | 1.767 | - | 2.445 |
| Iquipari | 1.178 | 1.035 | 1.112 | 1.717 | 1.429 | 1.475 | 2.445 | 0.849 | 1.221 |
| Massambaba | 0.895 | 1.042 | 1.442 | 2.294 | - | 2.181 | 1.273 | 1.944 | 1.621 |
| Maricá | 1.049 | 1.039 | 1.862 | 1.813 | 2.749 | 2.257 | 1.317 | 0.769 | 1.178 |
| Ilha do Cardoso | 0.613 | - | 1.365 | 1.095 | 1.691 | - | 3.110 | 2.377 | 0.689 |
| Dunas dos Ingleses | 1.353 | 1.225 | 1.839 | 1.488 | 1.186 | 2.353 | 1.362 | 1.731 | 1.835 |
| Dunas de Joaquina | 1.316 | 0.629 | 0.859 | 0.913 | - | 1.759 | 1.565 | 1.460 | 0.643 |
| **Spiders** | | | | | | | | | |
| Salvador | 1.077 | 1.657 | 0.659 | 1.472 | - | 2.011 | 0.695 | 0.888 | 1.167 |
| Trancoso | 2.134 | 2.282 | 0.976 | 1.121 | - | 1.121 | 1.624 | 1.855 | 1.604 |
| Barra Nova | 2.271 | - | 1.492 | 1.062 | 2.888 | 0.747 | 1.515 | 0.641 | 1.861 |
| Setiba | 9.424 | - | 2.681 | 0.727 | 2.813 | 1.943 | 1.838 | 1.228 | 1.16 |
| Praia das Neves | 3.518 | 4.809 | 1.988 | 0.608 | - | 3.583 | 1.521 | 3.511 | 1.622 |
| Iquipari | 1.826 | 3.235 | 0.825 | 1.199 | 2.902 | 1.314 | 1.431 | 1.950 | 1.932 |
| Massambaba | 2.207 | 1.264 | 2.187 | 0.886 | - | 1.793 | 1.326 | 1.288 | 1.095 |
| Maricá | 1.770 | 1.268 | 0.723 | 0.659 | 1.466 | 2.085 | 1.444 | 1.094 | 1.665 |
| Ilha do Cardoso | 1.622 | 1.356 | 0.877 | 1.316 | - | 1.239 | 1.574 | 1.111 | 1.622 |
| Dunas dos Ingleses | 3.727 | 2.202 | 2.540 | 0.738 | - | 4.842 | 1.927 | 1.446 | 1.716 |
| Dunas de Joaquina | 3.153 | - | 1.682 | 1.125 | 3.016 | 2.697 | 1.543 | 1.414 | 1.589 |
